# Prosthetic Valve Endocarditis by *Acinetobacter baumannii:* Case Report and Systematic Descriptive Review

**DOI:** 10.3390/pathogens15060581

**Published:** 2026-05-28

**Authors:** Annabella Salvati, Loredana Alessio, Gloria Trombaccia, Giovanni Cimmino, Marisa De Feo, Fausto Ferraro, Stefania De Pascalis, Nicola Coppola

**Affiliations:** 1 Infectious Diseases Unit, Department of Mental Health and Public Medicine, University of Campania Luigi Vanvitelli, 80131 Naples, Italy; annabella.salvati@studenti.unicampania.it (A.S.); loredana.alessio@gmail.com (L.A.); stefydps@gmail.com (S.D.P.); 2 Internal Medicine Unit, “Sacro Cuore”–Fatebenefratelli Hospital, 82100 Benevento, Italy; trombacciagloria@gmail.com; 3 Cardiology Unit, Azienda Ospedaliera Universitaria Luigi Vanvitelli, 80138 Napoli, Italy; giovanni.cimmino@unicampania.it; 4 Department of Women, Child and General and Specialized Surgery, Section of Anesthesiology, University of Campania Luigi Vanvitelli, 80131 Naples, Italy; marisa.defeo@unicampania.it; 5 Department of Translational Medical Sciences, Section of Cardiac Surgery and Heart Transplant, University of Campania Luigi Vanvitelli, 80131 Naples, Italy; fausto.ferraro@unicampania.it

**Keywords:** *Acinetobacter baumannii*, extensively drug-resistant bacteria (XDR), infective endocarditis, prosthetic valve endocarditis, antimicrobial resistance, osteomyelitis, cefiderocol, surgical management

## Abstract

A 61-year-old woman developed prosthetic valve infective endocarditis after osteomyelitis caused by extensively drug-resistant (XDR) *Acinetobacter baumannii*. Moreover, a systematic descriptive review of published case reports was performed to describe the main features, treatment, and outcomes of this condition. Methods: Apart from the description of our case, a PubMed and Embase literature review was conducted up to January 2026 using the terms “*A. baumannii*” AND (“infective endocarditis” OR “endocarditis” OR “valvular infection”). We included clinical cases of IE caused by *A. baumannii* published as full-text articles in English. Results: After orthopedic osteosynthesis surgery following a femur fracture, our patient developed osteomyelitis by XDR *A. baumannii* and was treated for a short period of time. Later, prosthetic aortic valve endocarditis was diagnosed. Despite treatment with cefiderocol and eravacycline, she died. An additional 18 clinical cases of IE by *A. baumannii* were identified from the literature, bringing the total to 19 cases. IE affected prosthetic valves in nine cases, native valves in nine and involved a right atrial mass in one. Twelve cases were caused by MDR or XDR *A. baumannii*. Mortality occurred more frequently in cases not treated with surgery (9/13, 69%) compared to those treated with surgery (1/6, 16.7%). However, given the very small sample size, these data should be interpreted with caution. Conclusion: This case, together with previously reported observations, highlights the severity of EI by *A. baumannii* and the need of multidisciplinary management.

## 1. Introduction

Infective endocarditis (IE) is a rare, but clinically significant condition; in 2019, the estimated incidence of IE in the general population was 13.8 cases per 100,000 subjects per year, accounting for 66,300 deaths worldwide [1]; however, its incidence is significantly higher in patients with prosthetic heart valves (exceeding 4 per 1000 patient-years) and in those with a history of previous endocarditis (exceeding 10 per 1000 patient-years) [2]. Considering its high morbidity and mortality—1723.59 disability-adjusted life years (DALYs) and 0.87 deaths per 100,000 population—rapid diagnosis and appropriate therapy remain essential [3].

The portals of entry for pathogens include skin infections, oral cavity, respiratory, gastrointestinal, or genitourinary tracts; direct inoculation occurs in people who inject drugs (PWID) and healthcare-related exposures, such as invasive diagnostic or surgical procedures [4,5]. Patients with prosthetic or repaired cardiac valves are at higher risk of IE after invasive procedures.

The most common causative microorganisms of IE are Gram-positive cocci, including *Staphylococcus*, *Streptococcus*, and *Enterococcus* species. *Staphylococcus aureus* predominates in prosthetic valve endocarditis, whereas viridans group streptococci are more frequent in native-valve disease. Less commonly, Gram-negative bacilli—including the HACEK group (i.e., *Haemophilus* spp., *Aggregatibacter actinomycetemcomitans*, *Cardiobacterium hominis*, *Eikenella corrodens*, and *Kingella kingae*), which are part of the normal oral and upper respiratory tract flora—as well as fungi and fastidious organisms such as *Coxiella burnetii*, may be responsible [6,7].

Among non-fermenting Gram-negative organisms, *Acinetobacter baumannii* is an important nosocomial pathogen that causes a range of healthcare-associated infections, including pneumonia, bloodstream infections, urinary tract infections, and wound infections, particularly in intensive care unit (ICU) patients. Although largely associated with hospital environments, extra-hospital reservoirs such as soil, water, animals, and food have also been documented [8,9]. While community-acquired strains often remain susceptible to carbapenems, healthcare-associated strains are frequently multi-drug resistant, especially carbapenem-resistant strains, limiting therapeutic options and increasing mortality. Accordingly, the World Health Organization has classified carbapenem-resistant *A. baumannii* as a “critical” priority pathogen [10]. To date, cases of IE caused by *Acinetobacter* spp. are rare and poorly described. We report a case of prosthetic aortic valve IE by extensively drug-resistant (XDR) *A. baumannii*—defined as susceptible to only one or two antimicrobial classes, excluding carbapenems—secondary to postoperative osteomyelitis. We also provide a systematic descriptive review of all published cases of IE by *A. baumannii* to date.

## 2. Case Description

A 61-year-old woman with history of mechanical aortic valve replacement (SORIN BICARBON 21 mm) in 2020, on chronic warfarin therapy, was admitted to a peripheral hospital on 3 January 2025, for a femoral fracture. She underwent surgical osteosynthesis with intramedullary nail placement the following week, receiving cefazolin prophylaxis. Table 1 shows the patient’s clinical history.

Three weeks later, purulent discharge developed at the surgical site. A tissue swab collected on 18 February 2025 grew *A. baumannii*, susceptible only to colistin.

(Table S1). Computed tomography (CT) scan was performed and showed soft tissue swelling, fluid collection and sinus tract formation which required surgical debridement and removal of the intramedullary nail. Intra-operative cultures confirmed *A. baumannii*, so she received standard-dose colistin for 7 days after surgical debridement, with an apparent improvement of her clinical condition.

However, on 17 March 2025—about two months after the initial surgery—she developed fever and signs of sepsis. Magnetic resonance imaging (MRI) revealed acute diffuse osteomyelitis with cortical bone destruction at the basi-cervical region and near the greater trochanter, classified as grade IV according to the Cierny–Mader system [11]. Growth of *A. baumannii* was obtained from three different blood cultures set, collected at three different times (Table S1). Targeted antimicrobial therapy was started with cefiderocol (2 g every 8 h) and fosfomycin (16 g/day) for its anti-biofilm activity. The fever resolved and the follow-up blood cultures performed on March 21 were negative. Moreover, during hospitalization, the patient developed *Clostridioides difficile* colitis, treated with fidaxomicin.

On March 31, she was transferred to our Infectious Diseases Unit for management of osteomyelitis, to perform a bone biopsy and reassess antimicrobial therapy.

On 6 April 2025, fosfomycin was discontinued and replaced with ampicillin/sulbactam (total daily sulbactam dose: 9 g), due to severe hypernatremia, while cefiderocol was continued. On 16 April 2025, transthoracic echocardiography (TTE) revealed vegetations on the prosthetic aortic valve, with a slight increase in the transvalvular gradient (mean gradient 15 mmHg, Vmax 2.7 m/s) and anterior leak of at least moderate grade in the mechanical prosthesis.

On 18 April 2025, whole-body 18F-FDG PET-CT (18-fluorodeoxyglucose-positron emission tomography/computed tomography) showed increased tracer uptake along the entire aortic valvular plane, consistent with active infection. Thus, based on the presence of multiple (more than three) positive blood cultures and imaging criteria (transthoracic echocardiography and PET-CT findings), the case met the modified Duke’s criteria (two major criteria) for *A. baumannii* IE [12]. Transesophageal echocardiography was deemed unnecessary.

To provide more active antibacterial treatment, eravacycline, as the newest available therapeutic option, was introduced in combination with cefiderocol (2 g every 8 h). Although the Multidisciplinary Endocarditis Team convened and recommended valve replacement along with targeted combination antimicrobial therapy, in accordance with ESC guidelines, surgery was ultimately deemed unfeasible due to the patient’s progressive clinical deterioration, including severe hypotension and anuric renal failure, which resulted in an unacceptably high surgery risk. She rapidly progressed to refractory septic shock and died on 10 May 2025.

The patient’s family informed consent was obtained for the publication of the clinical case, and the study was approved by the Ethics Committee of the University of Campania L. Vanvitelli, Naples (n°17122/2023).

## 3. Systematic Review of Literature

A systematic descriptive literature review of published case reports was conducted in line with the Preferred Reporting Items for Systematic Reviews and Meta-Analyses (PRISMA) 2020 guidelines [13]. The protocol was registered on PROSPERO database (PROSPERO registration number: CRD420261339133).

A literature search was performed in PubMed and Embase for original reports published as full papers from inception to 31 January 2026.

The PICO question used was:

Population (P): Adult patients diagnosed with infective endocarditis caused by *A. baumannii*, involving either native or prosthetic cardiac valves.

Intervention (I): Antibiotic treatment and/or surgical valve replacement or cardiac surgery performed as part of infective endocarditis management.

Comparison (C): No formal comparator group was defined due to the descriptive nature of the available literature.

Outcome (O): Primary outcome: Mortality; Secondary descriptive outcomes: Type of valve involvement (native vs. prosthetic), antimicrobial therapy administered, acquisition setting (ICU vs. non-ICU), and presence of comorbidities or risk factors.

### 3.1. Search Strategy

The search strategy was developed using a combination of free-text terms and controlled vocabulary related to *A. baumannii* and infective endocarditis or valvular infections. The following terms were combined using Boolean operators: “*A. baumannii*” AND (“infective endocarditis” OR “endocarditis” OR “valvular infection”)

For PubMed, Medical Subject Headings (MeSH) such as “*Acinetobacter baumannii*” and “Endocarditis” were used when available. For Embase, the following Emtree terms were applied: (‘*Acinetobacter baumannii*’/exp OR ‘*Acinetobacter baumannii*’) AND (‘infective endocarditis’/exp OR ‘infective endocarditis’ OR ‘endocarditis’/exp OR ‘endocarditis’ OR ‘valvular infection’).

### 3.2. Eligibility Criteria

Studies were included if they met the following criteria: reported clinical cases of infective endocarditis caused by *A. baumannii;* provided sufficient clinical, microbiological, or treatment data to confirm the diagnosis; were full-text articles in English; and involved patients aged ≥18 years.

Exclusion criteria included secondary research papers (e.g., reviews, systematic reviews, meta-analyses), editorials and papers not reporting results on primary research; preclinical studies (in vitro or animal); studies not in English; cohort studies without specific data for *Acinetobacter* endocarditis; duplicated datasets; case reports involving microorganisms other than *Acinetobacter*; and case reports involving patients <18 years of age.

### 3.3. Study Selection Process and Outcomes

Two researchers (A.S., L.A.) independently evaluated the title and abstracts of all citations to select the articles to be included in the analysis, reporting the reasons for exclusion for each study. Thereafter, they retrieved the full texts of the previously selected articles to evaluate them for inclusion. In case of disagreement, a third author (N.C.) made the definite decision.

The primary outcome was the overall mortality. Demographic and clinical characteristics associated with *A. baumannii* IE, antimicrobial susceptibility, treatment type, and risk factors for mortality were evaluated as secondary outcomes.

### 3.4. Data Extraction and Definition

Data from each eligible study were extracted by two investigators (A.S., L.A.). The extracted data included study type, year of publication and country, patient demographic data (age and gender) and patients’ relevant medical history (previous cardiac surgery or cardiac valve replacement, time after cardiac valve replacement), infection data and microbiology, treatment administered for IE, and outcomes (complications, cure or death). In case of disagreement, a third author (N.C.) made the definite decision.

### 3.5. Risk of Bias Assessment

The methodological quality of the included studies was assessed using the Joanna Briggs Institute (JBI) critical appraisal checklist [14]. Overall, the methodological quality was moderate to high, with scores ranging from 3/8 to 8/8. Most studies (73.7%) were classified as high quality, 10.5% as moderate quality and 15.8% as low quality (Table S2).

### 3.6. Statistical Analysis

Categorical variables were expressed as absolute or relative frequencies. Given the limited number of cases, all analyses were considered exploratory.

## 4. Results

**Figure 1:**
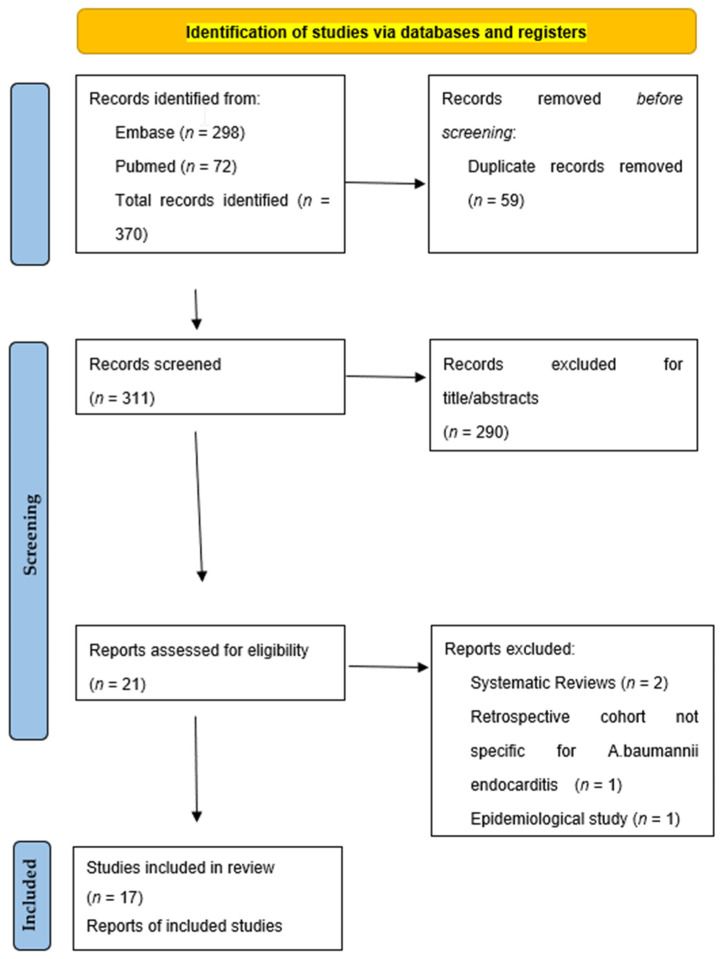
PRISMA Flow-chart.

An initial literature search yielded 298 studies on Embase and 72 on Pubmed, for a total of 370 articles (Figure 1). After removing 59 duplicates, 311 records were screened based on titles and abstracts. After title/abstract review 290 papers were excluded. After evaluating as full text, of the remaining 21 papers we excluded 4 additional studies because they did not meet the inclusion criteria. Thus, seventeen articles, reporting 18 cases, were included. In addition to our case, the final dataset comprised 19 cases [15,16,17,18,19,20,21,22,23,24,25,26,27,28,29,30,31].

Table 2 describes demographic, clinical, and microbiological characteristics, as well as the prognosis, of the clinical cases identified in the literature and of our case, for a total of 19 cases. Different predisposing factors were described, such as Intravenous Drug Users (IVDUs), valve replacement, previous surgery and endoscopy, though one subject had no known risk factors (Table 2). Notably, four patients (21.05%) had ICU hospitalization as a predisposing factor. However, except for two patients, all others had prior hospitalization. Five patients had undergone cardiac surgery (ranging from 11 years to 1 month before IE), three were on dialysis (from 2 months to 4 years), and one had colorectal surgery 2 months prior to IE diagnosis.

In nine cases (47.4%), endocarditis affected prosthetic valves (four mitral, three aortic, one tricuspid, one bivalvular aortic and mitralic). In nine (47.4%) cases, EI affected native valves (four mitral, two aortic, one tricuspid, one combined aortic and tricuspid involvement and one combined aortic and mitral valve involvement); one case (5.2%) manifested as a right atrial mass.

Regarding antimicrobial susceptibility, three cases (15.8%) were due to multi-drug resistant (MDR) *A. baumannii*, defined as resistant to at least three classes of antimicrobial agents—polymyxins, and tetracyclines/glycylcyclines, penicillins and cephalosporins, fluoroquinolones, and aminoglycosides [32]; nine cases (47.4%) were due to XDR strains [31] and five cases (26.3%) were due to fully susceptible strains. In two (10.52%) cases, antimicrobial susceptibility data were not reported.

Different antibiotic regimens were used in the 19 patients described, most of them in combination; in four cases antibiotic regimens were not reported (Table 2). Only six patients (31.6%) underwent surgical intervention.

Regarding the mortality, of the 19 cases evaluated, 10 patients (52.6%) died. The median age was similar between non-survivors (52.5 years, range 27–70) and survivors (56.0 years, range 26–74). Among the 10 patients who died, 5 (50%) had a prosthetic valve and an XDR *A. baumannii* strain; conversely, 44.5% of nine cases who survived had a prosthetic valve and an XDR *A. baumannii* strain. Death occurred in 9 (69%) of the 13 cases not treated with surgery and in 1 (16.7%) of the 6 treated. Moreover, comorbidities were observed in 8/12 non-surviving patients and in 2/7 in survivors. Thus, given the very small sample size, the present data should be interpreted with caution considering the analysis as exploratory.

## 5. Discussion

*A. baumannii* IE is a rare condition, with incidence estimates largely limited to case reports. A national Spanish prospective cohort reported an incidence of 2.9% for *Acinetobacter* IE among non-HACEK IE, with a higher mortality rate compared to endocarditis caused by other Gram-negative bacteria [33].

In the present paper, the clinical characteristics of 19 patients with IE by *A. baumannii* are described. To our knowledge, this is the largest description of patients with this specific infection. Although limited by the nature of a clinical cases review, it may assist clinicians in identifying risk factors, clinical characteristics, and management of this severe clinical disease. However, it seems reasonable to underline that the data from the present study should be interpreted with caution considering the exploratory nature of the analysis.

Regarding risk factors for infection, several have been identified in patients with *A. baumannii* infections other than IE. Examples include prolonged hospitalization (>90 days) or stay in intensive care units, advanced age, chronic comorbidities, invasive procedures or vascular access (such as central venous catheters or mechanical ventilation), and previous antibiotic therapy [34]. Of the 19 patients with *A. baumannii* IE evaluated in the present paper, only 4 patients had an ICU stay, but most had prior hospitalizations. Interestingly, in the presented clinical case, a postoperative orthopedic infection in a non-immunosuppresed patient represented the main risk factor, highlighting how lack of source control, also in low-risk subject, can also trigger IE by *A. baumannii*.

In regard to the clinical presentation, among the 19 cases described, IE involved native valves in nine patients and artificial valves in nine patients; in one case, the infection presented itself as right atrial mass. This underscores the pathogen’s ability to form biofilms on prosthetic material, contributing to persistence in healthcare settings and antibiotic resistance [35]. Recent investigations into medical implant infections have shown *A. baumannii* and stainless-steel implants as significant predictors of strong biofilm formation; furthermore, a significant relationship between MDR and biofilm production has been demonstrated [36]. Moreover, in our study, the reported patient presented with a sub-acute clinical course of IE, in contrast with the most previously reported cases that were diagnosed as acute IE, often associated with heart failure, or as post-mortem findings.

In regard to the microbiological finding, antimicrobial resistance in *A. baumannii* IE was very high. Of the 17 patients with available susceptibility data, 12 (70.6%) harbored MDR (3; 15.8%) or XDR (9; 47.4%) strains.

Management of *A. baumannii* infections, particularly those involving MDR/XDR strains and severe presentations such as IE, remains challenging. Antibiotic therapy is not standardized, and despite the introduction of novel antibiotics, overall clinical efficacy is often limited. While novel agents and combinations have shown promise in severe systemic infections, their role in deep-seated, biofilm-associated endocardial infection remains unclear.

Current therapeutic strategies include:

Cefiderocol: A siderophore cephalosporin with potent in vitro activity against many MDR and XDR *A. baumannii* isolates. Although European and American guidelines do not recommend the use of cefiderocol in infections produced by *A. baumannii* [37,38] unless no alternatives are available [38], recent data demonstrated a higher efficacy of cefiderocol than the best available therapy [39,40]. Still, evidence remains limited regarding efficacy in endocarditis, where vegetations pose a pharmacokinetic barrier. However, two clinical cases reported the use of cefiderocol in native aortic valve endocarditis by Pseudomonas aeruginosa and Achromobacter xylosoxidans, respectively, resulting in sterilization of both blood cultures and valve tissue [41,42]. Moreover, a possible advantage of cefiderocol lies in its activity against biofilm-forming Gram-negative pathogens; in fact, recent evidence suggested that this antibiotic may also help reduce already established biofilms produced by these bacteria [43].

Sulbactam–durlobactam: This combination targets β-lactamase-mediated resistance and has shown strong in vitro activity against *A. baumannii*, including carbapenem-resistant strains. In ATTACK trial sulbactam–durlobactam showed to be non-inferior to colistin-based therapy in patients with HAP, VAP or BSI [44]. Moreover, tissue penetration and activity against biofilm-embedded organisms remain areas of active investigation [45].

Given the paucity of monotherapy options, combination regimens are frequently deployed in clinical practice. These often include:

High-dose ampicillin–sulbactam (up to 9 g/day of sulbactam), leveraging intrinsic *Acinetobacter* spp. susceptibility, was recommended as backbone therapy in moderate/severe infections by American guidelines [37];

Colistin-based combinations: Evidence regarding colistin monotherapy versus combination therapy remains conflicting: despite concerns over nephrotoxicity and limited bactericidal activity, several studies have reported no differences in outcome (clinical cure and mortality) between patients treated with colistin monotherapy and those who received colistin in combination with meropenem, fosfomycin or rifampicin [46,47,48,49];

Tigecycline or eravacycline, which may offer broader Gram-negative coverage but present pharmacodynamic limitations in blood and heart tissue [50,51]. Precisely, eravacycline was approved in 2018 for treatment of complicated intra-abdominal infections (cIAIs) following the IGNITE1 and IGNITE4 trials exclusively focused on the infection site rather than the causative organism [52,53]. However, no data is available on the role of eravacycline in the treatment of endocarditis.

While in vitro synergy has been reported for specific combinations, clinical confirmatory evidence remains largely retrospective and observational. In endocarditis, where high bacterial loads and vegetative structures challenge drug penetration, the theoretical benefits of synergistic combinations must be weighed against toxicity and resistance development [54,55].

In this case, for the first time, new antibiotics—cefiderocol and eravacycline—were used to manage *A. baumannii* XDR IE.

The available literature is largely limited to case reports and small case series, with no randomized controlled trials. There are no dedicated guidelines for this specific entity; existing recommendations primarily address CRAB bacteremia and other invasive infections (e.g., HAP/VAP). Consequently, the overall level of evidence is very low and relies mainly on expert opinion and real-world data.

Regarding the prognosis, although specific data on *A. baumannii* IE mortality are scarce, a study about bloodstream infections caused by carbapenem-resistant *A. baumannii* in Italy showed 14-day and 30-day mortality of 61.2% and 73.6%, respectively [56]. Another review about *A. baumannii* infection-related mortality in hospitalized patients showed a mortality of 40%, with advanced age, admission to the critical care unit, male gender, comorbidities, prolonged hospitalization (>7 days), and hospital-acquired infection acting as significant mortality predictors [57].

In the present study, overall mortality was 52.6% with no difference between valve types or antimicrobial susceptibility of the strain. Mortality occurred in 9/13 cases (69%) among patients who did not undergo surgery and 1/6 (16.7%) among those who did. However, the presence of comorbidities was observed in 8/12 non-surviving patients and in 2/7 in survivors, suggesting a potential selection bias in surgery treatment allocation. These observations came from a limited number of clinical cases (*n* = 19) and should be interpreted with caution, in severe infections caused by highly resistant strains such as *A. baumannii* IE.

The study has some limitations. First, the nature of the study did not allow for the exclusion of bias. Second, the number of cases evaluated was very low, and thus, conclusive statements regarding the management of *A. baumannii* IE were not possible. Third, data on the antimicrobial susceptibility of the isolated *Acinetobacter baumannii* strains and the rationale for the chosen antimicrobial therapy were not always available.

## 6. Conclusions

Despite the limitations of the study, our findings regarding *A. baumannii* IE highlight the importance of early diagnosis and individualized multidisciplinary management rather than supporting a specific therapeutic strategy. However, further studies will be required to better characterize antimicrobial treatment strategies and the role and timing of the surgery for *A. baumannii* infective endocarditis.

## Figures and Tables

**Table 1:** Clinical, microbiological and biochemical data of the clinical case.

	10 January 2025	18 February 2025	18 March 2025	21 March 2025	6 April 2025	24 April 2025	10 May 2025
Antibiotic treatment	Surgical osteosynthesis	Colistin	Cefiderocol + Fosfomycin	Cefiderocol + Fosfomycin Fidaxomicin	Ampicillin/Sulbactam 9 g + Cefiderocol	Cefiderocol + Eravacycline	Cefiderocol + Eravacycline
Clinical event	Fracture	Surgical Site Infection	Sepsis	C.difficile diarrhea	Prosthetic aortic valve IE	Fever	Death
Fever	No	Yes	Yes	No	Yes	Yes	No
Cultures for *A. baumannii*	Negative	Positive from Tissue swab	Positive from blood	Negative from blood	Negative from blood	Negative from blood	Negative from blood
White blood cells/uL	9 × 10^3^	10.1 × 10^3^	9.2 × 10^3^	10.2 × 10^3^	5.62 × 10^3^	5.99 × 10^3^	4.81 × 10^3^
Neutrophils	80%	82%	85%	85.5%	75.3%	70%	90.5%
C-reactive protein (mg/dL)	2.5	2.8	3.0	3.7	1.27	1.41	1.32
Creatinine (mg/dL)	1.2	1.3	1.8	2.37	1.36	1.21	2.67

**Table 2:** Demographic, clinic and microbiological characteristics of our cases reported in the literature.

Author (Year)	Age	Sex	Predisposing Factor	ICU-Acquired	Valve Involved	Embolic Events	MDR/XDR	Antimicrobial Therapy	Surgery	Outcome
Current case	61	F	Orthopedic surgery	No	Prosthetic aortic	Yes	XDR	CEF + FOS	No	Death
Baghan-Bruno (2010) [15]	26	F	Bariatric surgery; hemodialysis (FSG)	No	Right atrium mass	No	No	CFPM + VCM + GENT +	Yes	Alive
Chen (2015) [16]	56	F	Previous antibiotic exposure	No	Bioprosthetic tricuspid	No	XDR	3GC + SAM	Yes	Alive
Cheng (2019) [17]	38	M	IVDU	No	Natural tricuspid	Yes	No	MEM	No	Alive
Kica (2013) [18]	65	F	Cardiothoracic surgery	No	Prosthetic mitral	No	No	PIP/TAZ + CIP	No	Alive
Kunhi (2016) [19]	39	M	Hemodialysis	Yes	Aortic root abscess; native aortic + mitral	No	XDR	COL+ LIN	Yes	Alive
Laganà (2015) [20]	69	F	CABG + MVR	NK	Bioprosthetic mitral	No	XDR	Not specified	No	Death
Laganà (2015) [20]	70	M	MVR	NK	Bioprosthetic mitral	No	XDR	Not specified	No	Death
Lahmidi (2020) [21]	54	F	Intravascular catheter	Yes	Native mitral	No	MDR	COL	No	Death
Menon (2006) [22]	27	F	Surgery for aneurysm	NK	Native aortic	NK	MDR	CFPM + GENT	No	Death
Olut (2005) [23]	45	F	Cardiothoracic surgery	Yes	Prosthetic aortic	Yes	MDR	LVF + NET	No	Death
Patel (2015) [24]	51	M	Kidney–pancreas transplant	No	Native mitral	Yes	XDR	Not specified	No	Death
Qureshi (2022) [25]	38	F	None	No	Native aortic + tricuspid	No	XDR	COL	Yes	Death
Rizos (2007) [26]	67	M	Cardiothoracic surgery	No	Prostetic aortic	No	No	MEM + TOB	Yes	Alive
Rosa (2013) [27]	39	F	Hemodialysis	No	Native mitral	No	XDR	CEF + TOB	No	Alive
Shokouhi (2021) [28]	73	M	Biliary surgery	No	Native aortic	No	XDR	COL + TYG	Yes	Alive
Sturiale (2014) [29]	69	F	CABG + MVR	Yes	Bioprosthetic mitral	No	Not specified	Not specified	No	Death
Yu-Hsien (2008) [30]	74	M	RHD + colonoscopy	No	Bioprosthetic mitral	No	No	3GC + AMP/SUL	No	Alive
Yadav (2022) [31]	36	M	ARP + EVAR + CABG + Bentall	No	Native mitral	No	Not specified	3GC + VCM	No	Death

M (Male), F (Female), CABG (Coronary Artery Bypass Graft surgery), IVDU (Intravenous Drug User), FSG (Focal Segmental Glomerulosclerosis), 3GC (3rd Generation Cephalosporines), PIP/TAZ (Piperacillin/tazobactam), CIP (Ciprofloxacin), SAM (Ampicillin/Sulbactam), COL (Colistin), GENT (Gentamycin), LVF (Levofloxacin), CFPM (Cefepime), CIP (Ciprofloxacin), AMP (Ampicillin), SUL (Sulbactam), MEM (Meropenem), VCM (Vancomycin), TOB (Tobramycin), CEF (Cefiderocol), TYG (Tygecicline), NET (netilmicin), LIN (Linezolid), FOS (Fosfomycin), (RHD (Rheumatic Heart Disease), MVR (Mitral Valve Replacement), AVR (Aortic Valve Replacement), ARP (Aortic Root Replacement), EVAR (Endovascular Aneurysm Repair).

## Data Availability

The data presented in this study are available on request from the corresponding author.

## Supplementary Materials

The following supporting information can be downloaded at: https://www.mdpi.com/article/10.3390/pathogens15060581/s1, Table S1: *Acinetobacter baumannii*’s susceptibility test according to EUCAST on tissue swab culture on 18 February 2025 and on blood cultures on 18 March 2025; Table S2: Methodological quality assessment using the Joanna Briggs Institute (JBI) critical appraisal checklist.
