# Supplementary material for: Prosthetic Valve Endocarditis by Acinetobacter baumannii: Case Report and Systematic Descriptive Review
Source: Pathogens. 2026 May 28;15(6):581. doi: 10.3390/pathogens15060581 (PMC13304811; doi:10.3390/pathogens15060581)
Supplement: Supplementary file 1 [file pathogens-15-00581-s001.zip › pathogens-4215197-supplementary.pdf]

**Antibiotic**	**MIC (ug/ml)**	**Interpretation**
Ampicillin/sulbactam	>32/16	R
Amikacin	>32	R
Ciprofloxacin	>2	R
Colistin	< 0.5	S
Gentamycin	>8	R
Imipenem	>8	R
Meropenem	>8	R
Meropenem/Vaborbactam	32	I
Piperacillin/Tazobactam	64	I
Tigecyclin	2	I
Tobramycin	>8	R
Cotrimoxazole	> 160	R

Supplementary Table S1. *Acinetobacter baumannii*'s susceptibility test according to EUCAST on tissue swab culture on February 18, 2025 and on blood cultures on March 18, 2025

Supplementary Table S2. Methodological quality assessment using the Joanna Briggs Institute (JBI) critical appraisal checklist

Study	Reference	Score	Percentage	Quality
Current case		8/8	100%	High
Baghan-Bruno (2010)	15	7/8	87.5%	High
Chen (2015)	16	8/8	100%	High
Cheng (2019)	17	7/8	87.5%	High
Kica (2013)	18	6/8	75%	High
Kunhi (2016)	19	7/8	87.5%	High
Laganà (2015)	20	3/7	42.9%	Low
Laganà (2015)	20	3/7	42.9%	Low
Lahmidi (2020)	21	7/7	100%	High
Menon (2006)	22	5/8	62.5%	Moderate
Olut (2005)	23	7/7	100%	High
Patel (2015)	24	5/8	62.5%	Moderate
Qureshi (2022)	25	8/8	100%	High
Rizos (2007)	26	8/8	100%	High
Rosa (2013)	27	7/8	87.5%	High
Shokouhi (2021)	28	8/8	100%	High
Sturiale (2014)	29	3/8	37.5%	Low
Yu-Hsien (2008)	30	7/7	100%	High
Yadav (2022)	31	8/8	100%	High
